# Data Integrity of Radiology Images Over an Insecure Network Using AES Technique

**DOI:** 10.31557/APJCP.2021.22.1.185

**Published:** 2021-01

**Authors:** Pavithra Prabhu, Manjunath K N, Chitra Rajarama, Anjali Kulkarni, Rajendra Rajendra

**Affiliations:** 1 *Department of Computer Science and Engineering, Manipal Institute of Technology, Manipal Academy of Higher Education, Manipal, India. *; 2 *Department of Information Science and Engineering, NIE Institute of Technology, Mysuru, India. *; 3 *Independent Consultant, AI in Radiation Oncology, Bengaluru, India. *; 4 *Research and Development, RTWO Healthcare Private LLP, Mahalakshmipuram, Bengaluru, India. *

**Keywords:** Digitally reconstructed radiograph, block cipher, AES technique, Zig-Zag pattern, encryption, decryption

## Abstract

**Background:**

While transmitting the medical images in radiology information systems the adversary effect can break the CIA (Confidentiality, Integrity, and Availability) triads of information security. The objective of the study was to transmit the complete set of image objects in a dataset without data integrity violation.

**Methods:**

In this paper a hybrid cryptographic technique which combines the prime details from the patient dataset (stack of axial 2D images) and the Advanced Encryption Standard (AES) method has been proposed. The steps include a) Creating an artificial X-ray image (DRR) from the 3D volume, b) dividing the DRR image in x and y directions equally into four regions, c) applying the zig-zag pattern to each quadrant, and d) encryption of each quadrant with block cipher mode using the AES algorithm. After dataset transmission the DRR image was regenerated at the receiver and compared each of the deciphered blocks (transmitted ones) using the histogram technique.

**Results:**

The technique was tested on CT and MRI scans of sixty datasets. The image injection techniques, such as adding and deleting an image from the dataset and modifying the image pixels, were tested. The results were validated statistically using mean square error and histogram matching techniques.

**Conclusion:**

The combination of the DRR and the AES technique has ensured the secured transmission of the entire dataset and not an individual image.

## Introduction

**Figure 1:**
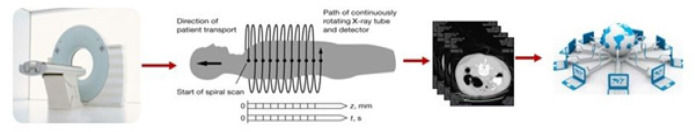
The Image Acquisition in Radio-Diagnosis Using the Spiral Scan Technology and Transmitting the Images Over the Network (CT scan of a patient including the image reconstruction from the projection data) (Image source: (Kalender, 2006))

Radiation Oncology workflow includes image acquisition of different modalities like CT, MRI and PET based on the clinical need. Post image acquisition, the images are transmitted from the PACS server to the workstation of the physicist for the treatment planning. When Radiologists have less time and the patients are more they analyze the images offline. Offline consultancy for analyzing images has been increasing in the recent past. The intra-hospital and inter-hospital networks access the images frequently and the data exchange is vulnerable. Image security in radiology software requires three main characteristics viz. Confidentiality, Integrity, and Availability (Ali et al., 2015; Anna and Sonal, 2015). In sequence they mean not disclosing the images to unauthorized persons, preventing the modification of the dataset and images should be accessible whenever the PACS server is queried. Usually, radiology image acquisition creates n number of images (in terms of hundreds to thousands). The number of images in a dataset mainly depends on the slice spacing, and the length of anatomy under scan in z-axis (SIEMENS CT, 2020), e.g., in CT Colonography case (Figure 1a, 1b), with ST={0.5mm, 0.625mm}, the image acquisition creates nearly three thousand images in both patient positions (prone and supine). 

The Radiologists analyses the images by accessing from the PACS server. The security of all the images of a dataset is most important when transmitted. These images are susceptible to attack by the adversary, and any image falsification by the adversary (I) in the network may mislead the image analysis by the radiologist. Many medical institutions and practitioners have implemented some of the mechanisms to protect the transmission of medical images. These mechanisms are a part of accepted standards including standards from DICOM, ISO, and IETF. At present the medical image transmission algorithms have adopted two significant methods – digital watermarking and cryptography to achieve the confidentiality and the integrity of the images. The objective of the study was to transmit the complete set of the image in a dataset without data integrity violation.

The detailed literature review is as follows. Dalel et al., (2011) discussed the checksum calculation by considering the header details at the CT image level. The sender calculates the checksum and appends the DICOM data and the receiver recalculates the checksum after transmission and compares both checksums. However, the complete transmission of the dataset was not guaranteed. Hiba and Ali (2017) discussed simultaneously embedding two watermarks in the spatial domain and encrypted domain. The algorithm implemented reversible data hiding on the histogram shift and assured the distortion-free images. 

To overcome the drawback of Kobyashi’s et al., (2009) technique, Ali and Noor (2015) discussed a method that generates symmetric keys and hash values. The drawback was only the pixels were encrypted and not the header. Natsheh et al., (2016) proposed an XOR based AES that encrypted both the pixels and the image header for the multi-frame DICOM images. From the collection of frames, the first encrypted frame was used as the reference to all other frames in the image. This reduced the computation time, ensuring confidentiality.

Puech and Rodrigues (2004) have implemented a crypto watermarking technique with both private and public keys and watermarking. It used the RSA algorithm and encrypted the image with the secret key and encryption of the secret key with the public-private key. This has a subtle disadvantage in generating the RSA key, which is computationally expensive for thousands of images in a dataset. While addressing confidentiality, Blackledge and Al-Rawi (2014) discussed a Stegacryption technique, which includes data encryption and hiding the encrypted data in the image using the Steganographic method. It removes private data before encryption.

Fathima and Chitra (2016) implemented a quaternation method with counter mode encryption and modular arithmetic operation, a time-efficient technique. Dalel et al., (2011) proposed the RC4 algorithm and Quantization Index Modulation (QIM) for watermarking the LSB of the image. It uses a set of codebooks based quantizers by inserting a message and quantifies the components of the image. In Suganya and Amudha, (2014), the authors implement the same method with RC4 algorithm.

Bhogal (2018) discussed encryption using AES and Arnold’s CATAES methods. Despite the results being accurate, the time taken to encrypt was longer when encrypting with CAT-AES due to the additional cat map step. There are a few other literatures: on watermarking techniques based on the DCT coefficients (Liao et al., 2015), ElGamal cryptosystem, and Elliptic curve algorithm (Dolendro and Manglem 2017), a blind digital watermarking technique (Nazir et al., 2018), image steganography (Muhammad, 2018) and using the logistic maps (Vibhor and Garima, 2018). Other methods like by embedding the DICOM attributes within the original axial slice to produce the watermarked image (Priya and Santhi, 2019), the encryption using the edge maps (Weijia, 2017), the application of DWT, DCT, and Elliptical Curve Diffie Hellman methods (Pooja et al., 2018), and adapting pixel thresholding approach (Qamar et al., 2018), a color medical image encryption which uses a chaotic map of two dimensional and Complemented-MLCPA (Hyun et al., 2018), dual encryption appositional based optimization algorithm (Avudaiappan, 2018) and symmetric encryption technique by Quist (Quist et al. 2015). The medical imaging standards DICOM from NEMA (2020) also has discussed the one-way hashing and the digital signature techniques in its security policies for image dataset transmission. Even though it is not computationally expensive the disadvantage is, if the attacker reveals the key, then the security is breached.

Regardless of well-established encryption schemes and secured image transmission we hardly found literature that discussed the entire dataset transmission as most of the methods were implemented at the individual image level. This is highly desirous, as there can be image series of the same modality or a different modality, RT objects, structure sets, and scout images within a dataset. The objective of the proposed work is to assure dataset integrity by combining the image processing method and the AES encryption scheme. 

We got CT and MRI datasets of head and neck cancer, prostate tumor, abdomen, CT Colonography, and nasopharynx from the Cancer Imaging Archive (the USA) website (TCIA, 2020). The CT scan parameters are 120kVp, 200mA, 512x512 image resolution, and ST of 1.0mm and 1.25mm. The scanners that acquired the image are from SIEMENS (2020), Philips (2019), and Toshiba (Toshiba, 2019). The work was implemented in C#.NET with Visual Studio 2012. The .NET framework supports the cryptography library. The DICOM image validation and the creation of DRR image functions have been considered from (Manjunath, 2017).

## Materials and Methods

**Figure 2:**
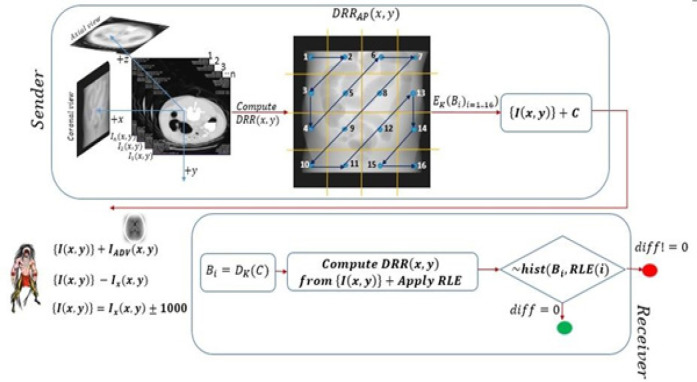
The DRR Image Calculation in a Specific Direction and Applying the Encryption Scheme on the Individual Blocks

The DICOM dataset usually contains images and non-image objects. Image objects include CT, MRI, PET modality images, and non-images includes the object notes, structured reports, registration and segmentation objects (and RT plans, fraction sequence objects in case of radiation therapy DICOM objects). Technically, the secured transmission includes transmitting both image and non-image objects. This work includes only image DICOM objects with a hybrid cryptographic technique. Security policies and mechanisms as defined in DICOM PS3.15 2020b (NEMA, 2020) standard (security and system and system management profiles), are the strong foundation for the proposed methodology. It comprises two phases (Figure 2): First, creating a DRR (Digitally Reconstructed Radiograph) image from the 3D volume, and then applying the cryptographic techniques on the DRR image. The CT image pixel stores Hounsfield Units (HU) in the scale of -1024 to+3072. Processing an image involves processing the HU and not the greyscale intensities. For display, greyscale intensities are derived from HU using window center (0028, 1050) and window width (0028, 1051).

*Step 1: Dataset validation*

Before the image validation, it is most important to check the data completeness in radiology images. Each image of the dataset is validated for type 2, type 1c, and type 1 attributes as per the DICOM standards PS 3.3. Type 1 attribute should have the tag as well as the value associated with it. In type 2 attribute, tag is mandatory but value is optional where in type 1c, value existence depends on the existence of type 1. Validation ensures the uniqueness and mandatory values of DICOM attributes. After this, the 2D slices are sorted as per the slice location (DICOM tag - 0020, 1041), and the 3D volume is reconstructed in a patient coordinate system (x, y, and z-axis) using linear interpolation technique to achieve isotropic voxels (Figure 2).

*Step 2: Creating a DRR image*

**Figure 3:**
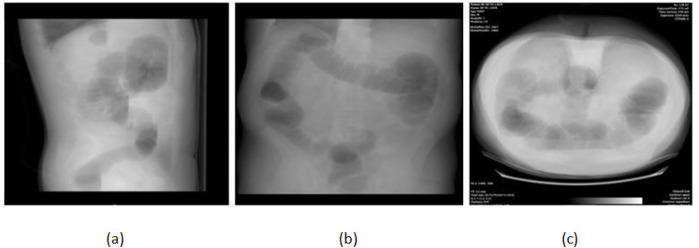
The 2D X-Ray Image Created from a Dataset of 1000 CT Images of Abdomen Scan which Shows the Large Intestine Distribution (C=-200, W=1500), A. Left-Right view, B. Anterior-Posterior view, and C. Inferior to Superior view

The DRR image in 2D is a recreated X-ray image from the 3D volume. This image shows the anatomy distribution of 3D volume on a 2D image. These images are created in a specific direction based on the clinical need to assess the anatomies. Figure 3 illustrates this (Anterior-Posterior in Figure 3B, Left-Right in Figure 3A and Superior-Inferior directions in Figure 3C). Compared to the axial view, the coronal and the sagittal view DRR image details are visually more appealing in displaying the anatomy. Coronal view DRR image is encrypted out of all three. Although the computation of DRR is expensive, the parallel computing of the DRR image pixel intensities at each location x,y has made the computation faster. The following pseudo-code illustrates computing the DRR image in a coronal direction.

// for each row in x-axis in 2D image

for r=0 to r<imageHeight;

// for each slice in the z-axis of the patient volume

for sliceBegin=zAxisBegin to zAxisEnd

// Summate the HU along the projection ray

CTNumbers.Add (HU[r,stackBegin + stackIndex,sliceBegin]);

// calculate the average

DRR [r,stackBegin + stackIndex] = CTNumbers.Average();

Similar to digital watermarking, the approach of fusing the DRR image to all the CT images individually and encrypting the image pixels would give better results as DRR is generated and fused its pixels from and to the same dataset respectively. When the adversary attempts to decode the key to decrypt the cipher, and even if he succeeds, it is very difficult to decode the logic of how the image pixels are modified. This approach is computationally inefficient.

*Step 3: Applying a zig-zag pattern for blocks*

The blocks are scrambled by applying the zigzag pattern on sixteen quadrants of the DRR image. The zig-zag pattern assures that the blocks are not sent in sequential order. It is difficult for an adversary to rearrange the blocks when sent in zig-zag order (whereas easy in the case when sent sequentially). The DRR divides into sixteen blocks (four rows and four columns) of the same size, i.e., blocks of size 128x128 (m/4*n/4) in the case of 512x512 dimension images and 64x64 of 256x256 size images. Usually, the medical images have a resolution of 512x512 (in CT modality), 256x256 (in MRI modality), and 1,024x1,024 (in case of high-resolution CT). The logic of 16 blocks of equal sizes works irrespective of the image dimension. There is no specific reason for following this 16 blocks pattern. For better accuracy, even the 64 blocks can also be considered. The example below shows the data structure and the sample values for storing the blocks. IDictionary is a container with key-value pair. The key and value are added or removed to the dictionary dynamically at runtime. The value can be a simple or a custom data type, such as a class or a structure.

IDictionary<int,int[,]>TheBlocks

Example:

TheBlocks [0] = {1,SubImage [Image.Rows/4,Image.Columns/4] }

TheBlocks [1] = {2,SubImage [Image.Rows/4,Image.Columns/4] }

TheBlocks [2] = {5,SubImage [Image.Rows/4,Image.Columns/4] }

Here, the dictionary index 0 corresponds to the first block of the image, the index 1 corresponds to the second, and index 3 refers to the fifth block in the zig-zag pattern. An image of 512x512 is divided into 16 quadrants with a block size of 128x128. This dictionary has the entries for 16 quadrants. Each of these blocks is encrypted in the next step.

*Step 4: The encryption algorithm*

Blocks from the results of step 3 are encrypted with the AES algorithm and user-defined key k. With this technique, it is hard for the adversary to know the algorithm used for encryption. The results of this step are stored in a dictionary, as shown below.

//Data structure

IDictionary<int,string>TheCipher

Int Counter=1;

//Converting each of the block (M) into cipher (C)

foreach Block_i_ in TheBlocks

{

 Counter++;

TheCipher.Add(Counter,AES(key, Block_i_) 

}

Example: 

TheCipher [0] = {1,”AAAA2SDSDOWOEW23O”}

TheCipher [1] = {2,”AAAA2TR2Y544FRRRTO1”}

The dictionary stores the block number and the ciphertext C. The sixteen encrypted blocks and the dataset are transmitted to the destination over a network.

*Step 5: Transmission*

The dataset, along with the cipher C (a list containing the ciphertext as string) of the DRR image is transmitted to the destination computer by assuming that the network is insecure, and there exists an adversary I. Testing the adversarial effects during the dataset transmission implements the following three test cases. These are the ideal cases of data integrity violations.

• By inserting a DICOM image I_ADV_ (x,y) to the dataset {I(x,y)} to be transmitted. Here, I_ADV_ can be either an image from some other dataset or the duplicated Image IOD (Information Object Definition) from the existing dataset itself.

• By deleting any image I_2_ (x,y) from the dataset {I(x,y)}

• By changing the pixels of any image I_2_ (x,y) from the set of images {I(x,y)} by updating its intensities randomly with f(x,y)±10, where 10 is the modified intensity value (HU).

In all cases, after generating DRR, the difference between the regenerated DRR and the original DRR is computed.

*Step 6: Decryption*

The receiver receives the dataset along with cipher C of the DRR image. The receiver decrypts the blocks with the key k (which was used for encryption at the sender). DRR is regenerated from the received dataset in anterior to posterior direction. The sixteen blocks of size 128x128 are extracted from this DRR using the zig-zag pattern. The histogram of these blocks of size 128x128 are compared one by one with the histogram of decrypted blocks (pseudo-code is shown below), and the root means square error (RMSE). If the difference of histograms of the DRR is equal to zero (i.e.~(hist_1_, hist_2_)), or if the RMSE is equal to zero, the data are preserved from the data integrity violation by I. In another way round, when the difference is greater than zero, it proves that the adversary has modified the dataset, which leads to failure in the comparison of the histogram of the DRR images. This failure is due to either the pixels are modified in the image or the addition of a new image I_ADV to the dataset {I(x,y)} or due to the removal of an image from the existing dataset.

bool comparison=true;

foreach(Block B inBlock_i_)

if hist(Block_i_) i=-1024…+3072 !=hist(Block_i_ (M))

 comparison =false;

After comparing all the blocks if the flag is true, assures the data integrity after the transmission. There may be chances where different patient datasets of the same anatomy result in similar DRR images. Therefore, histograms comparison at image level may fail. Hence, the difference in histograms of all 16 blocks is compared separately. Other methods such as Mean Absolute Error (MAE), Peak Signal to Noise Ratio (PSNR), and Normal Colour Distribution (NCD) could have been applied. The RMSE is calculated as the PSNR does not depend on image intensity. The PNSR measures the peak error, whereas the RMSE is the cumulative squared error between the two images. We also wanted to compare the pixel intensity between the two images at the specific geometrical location directly. Therefore, in addition to the RMSE, the histogram matching is also applied to crosscheck the results.

## Results

Empirically tested the proposed method on sixty datasets (n=60), including the CT and MRI images of the brain tumor, prostate cancer, abdomen, Nasopharynx, and CT Colonography. Each dataset had 100 – 2000 DICOM images per patient (scanned in both supine and prone positions). The number of images depends on the slice thickness selected during CT scan, and the length of the anatomy in z axis. The number of images in the dataset (as listed in Table 1) are, Colon CT1 = 1900, Ankle CT1 = 200, HeadNeck1 = 100, Nasopharynx = 150, Colon CT2 = 512, Colon CT3 = 2000 and HeadNeck.X = 90 slices. The image acquisition parameters of CT images are ST = {1.25mm, 2.5mm, 0.625mm}, kVp = {80, 100, 110}, mA = {200, 240, 300} and MRI images are, Scanning sequence = {SE, IR, EP}, Scan options = {PER, PFF, SP, RG}, MR acquisition type ={2D, 3D}, Imaged nucleus = {1H, 31P}, Image type = {MPR, T1 MAP, T2 MAP, PHASE MAP}, Spatial resolution = {1.67 – 1.99 mm}, Flip angle = {90, 85, 95, 110}.

**Figure 4:**
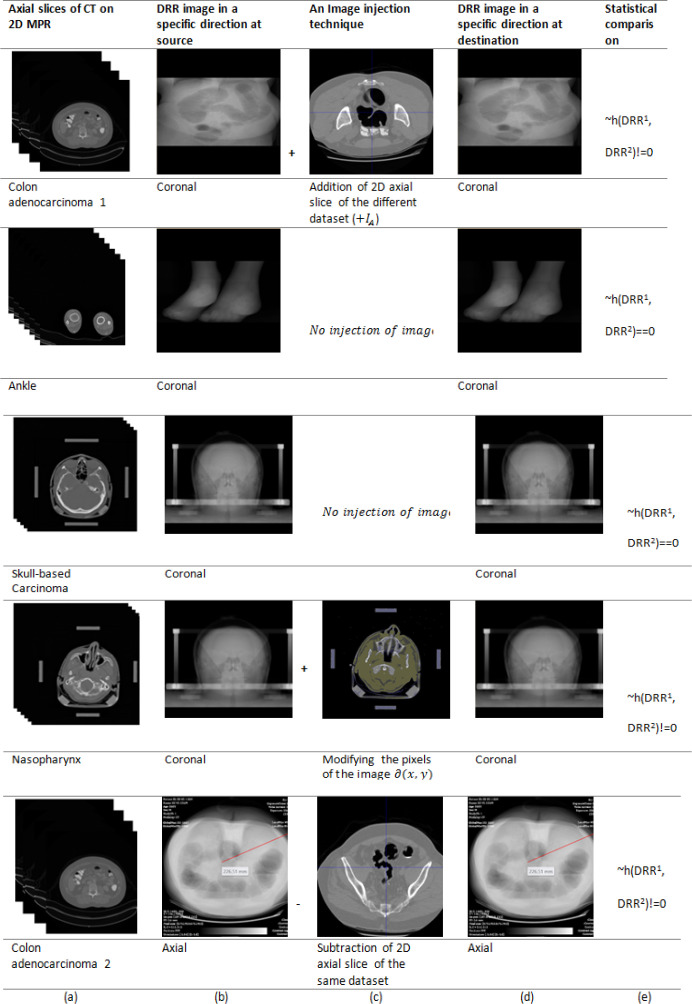
The Result of the Proposed Method Along with the Image Injection Techniques (addition - row 1, deletion - row 5, and the Modification of Pixel Intensities - row 4. Column (a): The stack of axial CT images of different datasets, Column (b): The DRR image created from the 3D volume. Column (c): Image injection techniques including the addition of image from other datasets, deletion from the existing dataset, and modifying the pixels. Column (d): The DRR image recalculated at the receiver. Column (e): The histogram difference of DRR of each block among sixteen blocks of an image at source and at the destination. Visualization of the images is with colon window settings (0C, 2000W)

Axial view DRR was extracted from ten datasets out of the sixty datasets, sagittal view DRR from fifteen, and the coronal view DRR from the rest of the datasets. Many medical image-processing applications validate the results qualitatively and quantitatively with the DRR image. The empirical testing includes injecting an external image to the dataset (Figure 4, row 1), deleting an existing image from the dataset (Figure 4, row 5), and modification of image pixels within the dataset (Figure 4, row 4). In rows 2 and 3 as there is no data modification the difference between the histograms of both the DRR results in the value zero. Before the image modification, name the DRR computed as DRR^1^(column 2), and after modification, named as DRR^2^ (column 2). 

As discussed in the previous section the DRR is compared at the block level after applying the zig-zag pattern. It could be possible that the adversary can modify the histogram of the image itself to fool the receiver and convince the receiver that there is no data modification. The blocks are sent in a zig-zag pattern to spoof the adversary. With any of the adversarial effects the block-wise comparison would fail at the receiver and the modification of the images could easily be identified. Since the DRR image pixels are computed directly from 3D volume data, a minor modification in the dataset reflects the intensity change in DRR pixels, and it leads toDRR_Bi_^1^ != DRR B_i_^2^ (column 5), where i is the corresponding block number. As humans we can identify only a few grey levels (16 levels) (Kalender, 2006), it is difficult to perceive the modified DRR shown in Column 4. The differences can be measured only through statistical comparison methods. The pixel-wise comparison, the histograms comparison, and the RMSE were employed to check the difference between DRR^1^ and DRR^2^. The difference of zero was noticed when the dataset was not modified. In a few datasets, the difference was greater than zero, which infers the data integrity violation. Our study did not check DICOM image header details, as many researchers have already implemented the checksum computation and comparison. 

**Table 1:** The Comparison of Different Metrics Calculated in the Empirical Testing (Only Ten Datasets are Shown)

			Use cases of image injection techniques			
Dataset	DRR direction	Metric	*+I*_A_	*-I*_A_	**∆** (*x,y*)	Inference
Colon CT1	Coronal	~hist(DRR^1^,DRR^2^)				data is modified
		RMSE	23.4	22.6	19.61	data is modified
Ankle CT1	Coronal	~hist(DRR^1^,DRR^2^)				data is modified
		RMSE	28.1	26.3	18.3	data is modified
HeadNeck1	Coronal	~hist(DRR^1^,DRR^2^)				data is modified
		RMSE	18.4	19.4	25.5	data is modified
Nasopharynx	Coronal	~hist(DRR^1^,DRR^2^)				data is modified
		RMSE	32.5	29.3	17.5	data is modified
Colon CT2	Axial	~hist(DRR^1^,DRR^2^)				data is modified
		RMSE	12.5	13.2	22.43	data is modified
Colon CT3	Axial	~hist(DRR^1^,DRR^2^)				data is modified
		RMSE	33.15	31.5	19.92	data is modified
HeadNeck2	Sagittal	~hist(DRR^1^,DRR^2^)				data is modified
		RMSE	24.65	26.33	22.12	data is modified
HeadNeck3	Axial	~hist(DRR^1^,DRR^2^)				data is modified
		RMSE	33.21	31	21.98	data is modified
HeadNeck4	Sagittal	~hist(DRR^1^,DRR^2^)				data is modified
		RMSE	22.1	23.4	14.33	data is modified
HeadNeck5	Coronal	~hist(DRR^1^,DRR^2^)				data is modified
		RMSE	45	41.5	9.4	data is modified

**Table 2:** Comparison of the Methodology and the Test Cases Reported in Other Literature

Authors	Methodology	Achieved confidentiality	Achieved Authenticity	Tested for DICOM image	Tested for dataset
Hiba and Ali 2017	Reversible watermarking technique	Yes	Yes	No	No
Fathima and Chitra, 2016	Quaternation technique + Countermode of encryption + DW T-SVD	Yes	Yes	Yes	No
Petros and mentos, 2016	ROI Reversible Steganography scheme	Yes	No	Yes	No
Anna and sonal 2015	Elgamal_Discrete Cosine transform function	Yes	Yes	No	No
Kobayashi et al., 2009	Hashing techniques	No	Yes	Yes	No
Ali et al., 2015	AES-Galois + Whirlpool + ECDSA	Yes	Yes	Yes	No
Sugnaya and Amudha 2014	AES+RC4+QIM	Yes	Yes	Yes	No
Blackleedge and al-Rawi, 2014	Stegacryption method	Yes	No	Yes	No
Dalel et al., 2011	RC4+LSB substitution+QIM	Yes	Yes	No	No
Puech and Rodrigues, 2014	Public Key and Secret key ciphering(RSA)+ Watermarking	Yes	Yes	No	No
Our study	AES encryption on DRR image	Yes	Yes	Yes	Yes

## Discussion

Table 1 shows the list of the datasets used for the empirical testing and the DRR image created in a specific axis in the patient coordinate system. The first five entries in the table correspond to the dataset, as shown in Figure 4. The next columns show the image injection techniques such as adding an image (+I_A_), and deleting an image (-I_A_) to and from a dataset and modification of pixel (∆(x,y)). If the entire dataset is replaced, including the ciphered blocks at the receiving end, it still indicates that the dataset is not modified, and this is a technical limitation.

This technique works on single frame DICOM images from volumetric acquisition (R^3^), where each object has separate headers. For the dataset in R^3^, we have derived the DRR image in R^2^ for encryption. The same logic holds good for the multi-frame DICOM objects. Further, for either single frame or multi-frame objects in a still higher dimension (let us say R^4^, R^5^), the DRR can be derived at one dimension lower than the dataset. As discussed in the literature review, to improve the security in addition to the proposed technique, we can apply the digital signature or hashing for non-image DICOM objects also. But these schemes have little drawbacks. In the case of the digital signature, the destination needs to have the digital signature of the sender stored in the local computer to unlock the signed images from the sender. At the moment, this is difficult as there is no clarity on how to transmit this signature to the destination, is it along with the dataset or as a separate entity. Also, some applications contact the third-party server (TTP) for the creation of the digital signature. In the case of the Hash function, the key used for creating the message digest has to be transmitted along with the message digest. Many state of the art techniques have implemented the hashing already. Also, the overhead of generating the DDR and sending this data, as well as processing on the receiving end behind the hospital firewall can slow down turn around on new cases as well as creates a considerable expense in processing power. But the little gain over current methods may provide better security.

The statistical measures, i.e., the RMSE calculated for each of the image injection techniques and the summation of the difference of histogram (of each block), are shown for ten datasets. The RMSE. For example, in Colon CT1, the difference of histogram is not zero; it infers that the dataset at the destination is modified. Only when there was no modification (in the case of AnkleCT1 and HeadNeck1), the RMSE and the difference of histogram is zero (infers data is secure). The AES has more resistance towards linear and differential cryptanalysis due to a better avalanche effect than the other techniques such as DES. The AES is much faster than the DES and is easy to implement on hardware. Since the AES supports the large key size than the other algorithms, it is less susceptible to the attacks from the adversaries, and with the use of precise block size, it is less susceptible to birthday attacks.

There are many object-based visualization techniques like maximum intensity projection, minimum intensity projection, DRR, and first voxel hit. Maximum intensity projection gives the details of only hard structures by sparing the tissue details. Minimum intensity projection gives only the dark to medium level Hounsfield intensities by sparing the hard structure details. The first voxel method gives only the surface details of the anatomy by sparing the internal details of anatomy. Compared to all these methods, the DRR gives all the details of the 3D volume irrespective of whether it is low intensities or high intensities from the given 3D volume.

The proposed technique can be applied directly on image modalities like CT, MRI, and PET etc. In case of non-image modalities of radiation oncology like RT Dose, RT Structures, RT Plan, Fraction Sequence objects as we cannot depend on image content directly, a Checksum can be calculated at the DICOM object level which is a representative candidate (an integer or float value) of the DICOM file. And this checksum can be encrypted separately and can be transmitted along with the RT Dose, RT Plans, and RT structure set. Ultimately a single parameter modification in these files by an adversary will lead to a mismatch in the checksum comparison when checksum1 and checksum2 are compared.

In the case of a 3D dataset, we can derive a 2D DRR as explained in this paper. In the case of a 4D dataset (respiratory motion cases), for each 3D dataset, separately 2D DRR images can be extracted. Either each of these 2D DRR can be encrypted or an average of the set of DDR images can be derived and can be encrypted separately. And the same logic is applicable for still higher dimensions. But the only concern is the computational cost which can be reduced with implementation on either CPU threads or GPU threads or a combination of both. This can be the scope of future work.

*Study limitations*

Empirical testing did not involve the real-time network environment. Instead, the adversary effects were modeled and checked through the client-server program. All the messages of the image transmission were logged for analysis. Thus, through the simulation environment, the robustness of the proposed technique is proved. Table 2 illustrates the comparison of the proposed work with other literatures. All other papers have tested their methods for secure transmission at the image level. The last column shows the testing of the encryption at the dataset level or the image level. Also, others have discussed only the authenticity and confidentiality and not the integrity of the image. The novelty of the proposed method has been proved through a statistical analysis since it is difficult to compare the accuracy and the efficiency of the proposed method with the existing work as the experimental setup and the dataset vary.

The state-of-the-art techniques in a secured medical image transmission have concentrated on CIA triads only at the DICOM image level in R^2^ dimension and not on the complete set of images of a dataset in R^3^. It is difficult to find the image injected in the dataset in the case of single image encryption. We have developed a more optimized encryption technique (AES) for the dataset in R^3^ dimension using the block cipher mode, and with the help of the DRR image, transmits the entire dataset more securely. The AES is not only faster than the DES but also easy to implement on hardware, and since it supports the large key size compared to the other algorithms, it is less susceptible to the attacks. The DRR image has been widely used in medical imaging applications to validate 3D volume. It is ideal to use the DRR to check the data integrity violation as it is recreated from the same dataset. Minor changes to the image pixels result in a change of DRR pixels. The failure in comparing the two DRRs at the sender and the receiver is the inference of dataset modification. We have considered only the single frame DICOM objects and not the multi-frame DICOM objects and non-image objects. This technique can be embedded with the image header to make the technique more robust.

Empirically tested the method on CT datasets with all adversarial acts, and detected the data integrity violations accurately. The technique can be extended further on to the images of MRI and the PET modalities also. The acquisition of higher dimension datasets (R^4^, R^5^) is usual in Radiology. The time complexity and the complexity in encryption increase with such datasets. Irrespective of the dimension, it is easy to apply the proposed method where accuracy is of concern. At present, the testing has been done only on one image series of the patient. The scope of the future work includes extending the same technique on multiple image series in the same dataset and multi-frame DICOM images in higher dimensions (R^4^, R^5^), embedding the DRR image pixels to the original set of images in the dataset, considering both image and non-image objects together while extracting the essential details for encryption and empirical testing in the real-time environment. Non-image DICOM objects are also essential to transmit, which are pertinent to the proper interpretation of the dataset

## Data Availability

The datasets analysed during the current study are available in the National Cancer Institute repository, https://public.cancerimagingarchive.net/ncia/login.jsf (http://doi.org/10.7937/K9/TCIA.2015.NWTESAY1)
